# Retraction Note: Identification of de novo mutations in prenatal neurodevelopment-associated genes in schizophrenia in two Han Chinese patient-sibling family-based cohorts

**DOI:** 10.1038/s41398-020-01116-6

**Published:** 2020-12-01

**Authors:** Shan Jiang, Daizhan Zhou, Yin-Ying Wang, Peilin Jia, Chunling Wan, Xingwang Li, Guang He, Dongmei Cao, Xiaoqian Jiang, Kenneth S. Kendler, Ming Tsuang, Travis Mize, Jain-Shing Wu, Yimei Lu, Lin He, Jingchun Chen, Zhongming Zhao, Xiangning Chen

**Affiliations:** 1 Center for Precision Health, School of Biomedical Informatics, The University of Texas Health Science Center at Houston, Houston, TX 77030 USA; 2 Bio-X Institutes, Key Laboratory for the Genetics of Developmental and Neuropsychiatric Disorders (Ministry of Education), Collaborative Innovation Center for Brain Science, Shanghai Jiao Tong University, Shanghai, China; 3 Shanghai Key Laboratory of Psychotic Disorders, Shanghai Mental Health Center, Shanghai Jiao Tong University School of Medicine, Shanghai, China; 4 School of Biomedical Informatics, The University of Texas Health Science Center at Houston, Houston, TX 77030 USA; 5 Virginia Institute of Psychiatric and Behavioral Genetics, Medical College of Virginia and Virginia Commonwealth University, Richmond, VA 23298 USA; 6 Department of Psychiatry, University of California at San Diego, San Diego, CA 92093 USA; 7 Department of Ecology and Evolutionary Biology, University of Colorado Boulder, Boulder, CO 80309 USA; 8 Institute for Behavioral Genetics, University of Colorado Boulder, Boulder, CO 80309 USA; 9 Nevada Institute of Personalized Medicine, University of Nevada Las Vegas, Las Vegas, NV 89154 USA; 10 Institute of Neuropsychiatric Science and Systems Biological Medicine, Shanghai Jiao Tong University, Shanghai, China; 11 MD Anderson Cancer Center UTHealth Graduate School of Biomedical Sciences, Houston, TX 77030 USA; 12 Human Genetics Center, School of Public Health, The University of Texas Health Science Center at Houston, Houston, TX 77030 USA; 13 410 AI, LLC, 10 Plummer Ct, Germantown, MD 20876 USA

**Keywords:** Schizophrenia, Genomics, Medical genetics

Correction to: *Translational Psychiatry*

10.1038/s41398-020-00987-z published online 1 September 2020

This article^1^ has been retracted at the request of Authors Xingwang Li and Lin He. After publication, it was realized that approval to use data from the NSFC-NIH Sino-US cooperation project (Project No. 81361120389) was not obtained from the data owners.

Authors Dongmei Cao, Xiangning Chen, Lin He, Kenneth Kendler, Xingwang Li, Travis Mize, Chunling Wan and Jain-Shing Wu agree to this retraction. Authors Shan Jiang, Jingchun Chen and Zongming Zhao do not agree to this retraction. Authors Guang He, Peilin Jia, Xiaoqian Jiang, Yimei Lu, Ming Tsuang, Yin-Ying Wang and Daizhan Zhou did not respond to correspondence from the Publisher about this retraction.
